# Molecular Detection and Genetic Characterization of Porcine Circovirus 2 (PCV-2) in Black-Backed Jackal (*Lupulella mesomelas*) in Namibia

**DOI:** 10.3390/ani12050620

**Published:** 2022-03-01

**Authors:** Umberto Molini, Lauren Michelle Coetzee, Leandra Van Zyl, Siegfried Khaiseb, Giovanni Cattoli, William G. Dundon, Giovanni Franzo

**Affiliations:** 1 School of Veterinary Medicine, Faculty of Health Sciences and Veterinary Medicine, Neudamm Campus, University of Namibia, Private Bag 13301, Windhoek 9000, Namibia; u.molini76@gmail.com (U.M.); leandravanzyl29@gmail.com (L.V.Z.); 2 Central Veterinary Laboratory (CVL), 24 Goethe Street, Private Bag 18137, Windhoek 9000, Namibia; laurenctz13@gmail.com (L.M.C.); khaisebs@gmail.com (S.K.); 3 Animal Production and Health Laboratory, Animal Production and Health Section, Department of Nuclear Sciences and Applications, Joint FAO/IAEA Division, International Atomic Energy Agency, P.O. Box 100, 1400 Vienna, Austria; g.cattoli@iaea.org (G.C.); w.dundon@iaea.org (W.G.D.); 4 Department of Animal Medicine, Production and Health, University of Padova, viale dell’Università 16, 35020 Legnaro, Italy

**Keywords:** porcine circovirus, PCV-2, jackals, Namibia, molecular epidemiology, genome

## Abstract

**Simple Summary:**

Although often considered host-specific, there is increasing evidence of a broader-than-expected host tropism for different circoviruses, including porcine circovirus 2 (PCV-2). In this study, the presence of PCV-2 in the lung lymph nodes of Namibian jackals has been demonstrated. The complete genome of the viruses was generated, and they were classified as PCV-2b and shown to have a close genetic relationship with South African and Namibian strains collected from domestic pigs. Since contact between jackals and domestic swine is highly unlikely, one of the most plausible explanations for the infection of the jackals with PCV-2b would involve the dispersal of pig-derived products in the wild environment during recreational activities or the scavenging activity of jackals living in peri-urban areas. However, further studies are required to properly assess how PCV-2 is acquired and maintained in the wild canids and its potential impact on other wild and domestic species.

**Abstract:**

Members of the genus Circovirus have been identified in several host species. While initially considered host-specific, there is growing evidence of their presence in unexpected hosts. Porcine circovirus 2 (PCV-2) is no exception, having been reported in animals belonging to different orders, including carnivores. Recently, PCV-2 was detected in domestic pigs, warthogs and antelopes in Namibia. Considering the potential contact between these populations and wild carnivores, the presence of PCV-2 was investigated in 32 black-backed jackals (*Lupulella mesomelas*) shot between February and July 2021 as part of predator control operations in Namibia. Two lung lymph nodes tested positive for PCV-2 by PCR, confirming the broader-than-expected PCV-2 host tropism and the susceptibility of canids. Sequence analysis demonstrated a close association between the PCV-2s identified in the jackals and PCV-2b strains collected from South African domestic pigs. Although several hypotheses regarding the source of the jackal’s infection are proposed, further studies should be performed to properly assess how PCV-2 is acquired and maintained in the wild and its potential impact on wild and domestic species.

## 1. Introduction

The genus *Circovirus* includes non-enveloped single-stranded ambisense DNA viruses with a genome of approximately 2kb, encoding two main proteins from two open reading frames (ORFs). ORF1 encodes the replication-associated protein Rep and Rep’ through alternative splicing, while ORF2 encodes the Cap protein, the only constituent of the viral capsid [1]. The Cap has been extensively studied, is responsible for viral attachment, contributing to host and cell tropism, and is the main target of the host immune response [2,3,4]. Moreover, because of its high genetic variability, the nucleotide sequence of ORF2 is used in molecular epidemiology studies [5,6]. Additionally, other ORFs and related proteins (ORF3-6) have been characterized and were proven to mainly be involved in apoptosis regulation and interaction with the host immune system [7,8,9,10].

Traditionally, circoviruses have been considered host-specific or restricted, at least, to a narrow host spectrum. For example, porcine circovirus type 2 (PCV-2), which causes several syndromes globally defined as porcine circovirus diseases (PCVD) and is responsible for major losses for the swine industry, was initially strongly associated with domestic pigs and wild boar [11,12,13,14]. However, since its identification in the late 1990s, evidence of PCV-2′s ability to infect distantly related species has emerged. Mice have been successfully used as experimental models [15,16] and, over time, other hosts have been reported to be infected with the virus, although the clinical and epidemiological relevance is much debated. For example, PCV-2 has been detected in cattle and calves [17,18,19] and rodents living near pig farms [20,21,22]. Even more surprisingly, some Chinese studies have reported PCV-2 infection in carnivores (i.e., mink, foxes, and raccoon dogs) in the presence of severe clinical signs [23,24,25].

Recently, the presence of PCV-2 was reported in Namibia, in domestic pigs, warthogs and Orynx antelope [26,27]. Due to the likely contact between these populations, especially warthogs and antelopes, and wild carnivores, the presence of PCV-2 in a jackal population in Namibia was investigated.

## 2. Materials and Methods

The study included lung lymph nodes from 32 black-backed jackals (*Lupulella mesomelas*) shot between February and July 2021 during predator control/culling operations (Table 1) in two farms with an area of about 10,000 hectares, each located in the Windhoek district, Khomas region, Namibia. All subjects were in good health prior to being shot and no clinical signs or lesions were observed. Samples were cooled on ice and then stored at −20 °C until processing on arrival in the laboratory.

The samples (50 mg of lung lymph node) were homogenized for 2 min in 1 mL of sterile phosphate-buffered saline (PBS) using a TissueLyser LT (QIAGEN, Hilden, Germany) and the DNA was extracted from 200 µL of each homogenate using the High Pure Viral Nucleic Acid Kit (Roche, Basilea, Switzerland) with an elution volume of 100 μL, as indicated by the manufacturer’s instructions. Four pairs of primers (Table 2) were used to detect the presence of PCV-2 and to generate an overlapping PCR-amplified covering for the entire genome.

The following thermal protocol was used: 94 °C for 5 min followed by 35 cycles of denaturation at 95 °C for 30 s, annealing at 51 °C for 30 s, and elongation at 72 °C for 60 s, followed by a final elongation at 72 °C for 5 min [28]. Amplicons of positive samples were purified using a Wizard SV Gel and PCR Clean-Up System (Promega, Madison, WI, US) and sequenced commercially by LGC Genomics (Berlin, Germany). The sequences of positive samples were submitted to the GenBank database under accession numbers OM417559-OM417560. The sequences were edited and assembled using the Staden software package version 2.0.0b8. Multiple sequence alignments were performed to compare and classify the PCV-2 sequences of the strains included in the study. More specifically, the full ORF2 reference dataset described by Franzo and Segalés (2018) [6] was downloaded and aligned to the ORF2 region of the sequences generated in this study at the codon level using the MAFFT method implemented in TranslatorX [29]. A maximum likelihood phylogenetic tree was reconstructed using IQ-Tree [30], selecting the model with the lowest Akaike information criterion as the best option. This was calculated using the same software. The robustness and reliability of the branching pattern was assessed by performing 10,000 ultrafast bootstrap replications. Moreover, to further contextualize the detected strains in the PCV-2 molecular epidemiology framework, a complete ORF2 dataset of strains whose collection country and date were available was downloaded from Genbank (accessed on 12 January 2022). Alignment and phylogenetic analyses were performed as previously described.

## 3. Results

**Figure 1:**
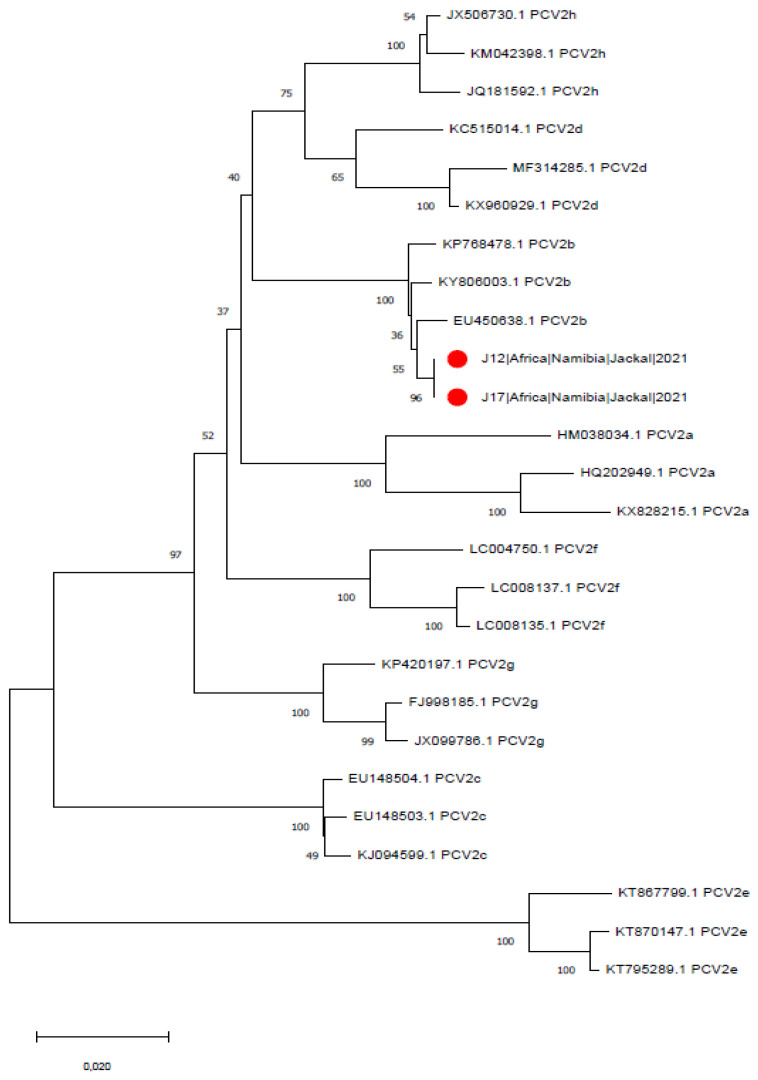
Maximum-likelihood phylogenetic tree based on complete ORF2 gene sequences of PCV-2 strains obtained in the present study (highlighted by red circles) and the reference sequences suggested by Franzo and Segalés (2018). The numbers nearby each node report the corresponding branch support.

Two out of 32 jackals (6.25%; 95CI: 0.77–20.81%) (Table 2), both from the same farm, tested positive for PCV-2 by PCR, and the complete genome was successfully sequenced. Comparison with the reference dataset of Franzo and Segalés (2018) allowed for its classification within the PCV-2b genotype (Figure 1).

**Figure 2:**
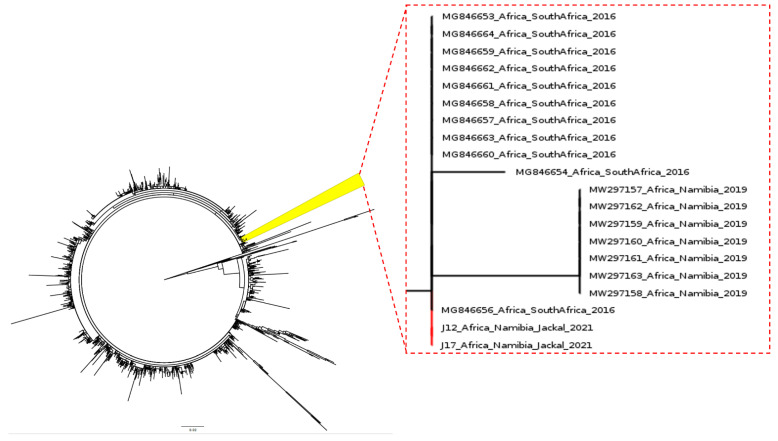
Maximum-likelihood phylogenetic tree based on a broad collection of complete ORF2 gene sequences of PCV-2b strains collected globally. The tree subset including the strains obtained in the present study (represented by branches) has been magnified (right insert).

The comparison of the ORF2 sequences from the jackal strains with a broad collection of PCV-2 ORF2 sequences for which collection date and country are available indicated a strong clustering with PCV-2b strains sampled in South Africa in 2016 (nucleotide identity of 100%) and, to a lesser extent (99.78% nucleotide identity), to Namibian PCV-2b strains from 2019 (Figure 2). Complete genome analysis revealed the closest identity (i.e., 99.88%) nucleotide identity with the South African strains.

## 4. Discussion

The present study reports the PCV-2 infection in Namibian jackals, with a non-negligible frequency.

The detection of circoviruses in different host species is not an unusual finding [31]. However, viral genome detection does not necessarily imply the susceptibility of the host, since the detection of the virus can often be explained by the ingestion of an infected meal, especially when feces, intestinal content or digestive organs are tested. However, the identification of PCV-2 in the lung lymph nodes of the jackals lessens the likelihood of this. Sample collection and processing was also performed according to good laboratory practices to minimize the risk of sample contamination. Therefore, the results of this study strongly support the broader-than-expected PCV-2 host tropism and the susceptibility of canids to this virus. In contrast to what has been previously reported regarding PCV-2 infection of carnivores [32], the subjects were in good health prior to being shot; no clinical signs or lesions were observed, suggesting an asymptomatic infection, although an incubation/convalescent carrier status cannot be excluded.

The ORF2 sequences analysis highlighted a strong clustering with PCV-2b strains sampled in South Africa in 2016 and, to a lesser extent, to Namibian PCV-2b strains from 2019, a finding confirmed by complete genome analysis.

The close association with viruses sampled in a neighboring country (i.e., South Africa) could suggest the circulation of such strains in the wild and their presence over a broad area. However, jackal populations are typically resident, and their generally limited movements are unlikely to cover, in this case, the large distance between the collection site and South Africa (>600 km). Moreover, the above hypothesis conflicts with the detection of PCV-2b strains in South African and Namibian domestic pigs, while warthogs and Oryx antelope were infected by PCV-2c [26], indicating that the PCV-2b in jackals may have originated from domestic pigs, even though direct contact between domestic pigs and jackals is thought to be highly unlikely. An alternative explanation could involve the dispersal of pig-derived products in the wild environment [33,34,35,36] during recreational activities or the scavenging activity of jackals living in peri-urban areas [37,38]. The presence and infectivity of PCV-2 in the meat and bone marrow of infected pigs has been demonstrated [39] and, combined with the high resistance of PCV-2 in the environment [40,41,42], could facilitate this infection path.

However, despite the importance of the current observations, and the increased knowledge of PCV-2 biology and epidemiology, further studies are required to confirm viral infection in newly identified hosts (e.g., demonstrating the viral/antigen presence in tissues/lesions by immunohistochemistry or in situ hybridization). In addition, how PCV-2 is acquired and maintained in the wild and its potential impact on wild species, including endangered ones, needs to be properly assessed.

## 5. Conclusions

The present study extends our knowledge of PCV-2 epidemiology and host tropism, demonstrating the occurrence of PCV-2 infection in carnivore wild populations, i.e., the Namibian jackals. The broad host tropism and plasticity of PCV-2 is, therefore, further supported. Further studies will be required to properly assess how PCV-2 is acquired and maintained in these wild canids and its potential impact on other wild and domestic species.

## Figures and Tables

**Table 1:** Description of the analysed samples.

Farm	Collecting Data	Number of Jackals	Age	Sex	PCR
A	20 February 2021	8	adult	F	-
			adult	F	-
			adult	M	-
			juvenile	F	+
			adult	F	-
			juvenile	M	-
			juvenile	F	-
			adult	F	+
	23 May 2021	2	juvenile	F	-
			adult	F	-
	11 July 2021	9	adult	M	-
			adult	M	-
			adult	F	-
			adult	M	-
			adult	F	-
			adult	F	-
			adult	F	-
			adult	F	-
			adult	F	-
B	23 May 2021	3	adult	F	-
			adult	F	-
			adult	M	-
	30 May 2021	4	adult	M	-
			adult	F	-
			adult	F	-
			adult	M	-
	13 June 2021	4	adult	F	-
			juvenile	F	-
			adult	F	-
			adult	M	-
	4 July 2021	2	adult	F	-
			adult	M	-

**Table 2:** Primers used in the study.

Primer	Sequences	Amplicon Size (bp)
P1	−5′-TAA TCC TTC CGA AGA CGA GC-3′	629
P2	3′-CGA TCA CAC AGT CTC AGT AG-5′	
P3	5′-CAG AAG CGT GAT TGG AAG AC-3′	630
P4	3′-ATG TAG ACC ACG TAG GCC TC-5′	
P5	5′-AGA AGC TCT TTA TCG GAG GA-3′	701
P6	3′-AAG CGA ACC ACA GTC AGA AC-5′	
P7	5′-CTA GAA TAA CAG CAC TGG AG-3′	621
P8	3′-GTT CGT CCT TCC TCA TTA CC-5′	

## Data Availability

The data that support the findings of this study are openly available in GenBank.
